# ‘I Wish It Were More Often Told to People Before They Are Prescribed These Medications How Hard It Is to Get Off Them’: A Qualitative Descriptive Analysis of Free‐Text Responses to a Survey on Reducing and Stopping Psychiatric Medication

**DOI:** 10.1111/hex.70384

**Published:** 2025-08-11

**Authors:** Miriam Boland, Agnes Higgins, Sookyung Kwak, Cathal Cadogan

**Affiliations:** ^1^ School of Pharmacy and Pharmaceutical Sciences Trinity College Dublin Dublin Ireland; ^2^ School of Nursing and Midwifery Trinity College Dublin Dublin Ireland

**Keywords:** antidepressants, antipsychotics, benzodiazepines, discontinuation, psychiatric, qualitative descriptive analysis, tapering, withdrawal

## Abstract

**Introduction:**

Despite significant increases in the prescribing of psychiatric medication in recent years, many uncertainties exist regarding the process of reducing and stopping these medications. A James Lind Alliance Priority Setting Partnership (PSP) study was conducted to identify the top 10 research priorities on reducing and stopping psychiatric medication. As part of the PSP study, an online survey was conducted, which asked respondents to submit their views and experiences of reducing and/or stopping psychiatric medication as free‐text comments. This study aimed to conduct a descriptive analysis on these free‐text survey responses.

**Methods:**

A qualitative descriptive analysis was undertaken on responses submitted to the online survey, which was disseminated using social media, newsletters and emails. Responses were submitted by three stakeholder groups (i.e., people with lived experience of taking and/or stopping psychiatric medication, family members/carers/supporters and healthcare professionals). All survey responses were downloaded, screened and analysed using template analysis. A coding template was iteratively developed using samples of responses and then applied to the responses by the researchers working independently.

**Results:**

In total, 705 responses contained free‐text additional comments, of which 483 responses were considered in‐scope. Six main themes were identified: (1) experiences of psychiatric medication, (2) challenges to reducing/stopping psychiatric medication, (3) strategies used to reduce/stop psychiatric medication, (4) outcomes of reducing/stopping psychiatric medication, (5) emotional context and (6) areas for improvement.

**Conclusion:**

This study identified numerous challenges faced by respondents when discontinuing psychiatric medication, the uncertainty that prevails in terms of the best tapering strategy and the emotional impact of taking and/or stopping psychiatric medication. The findings also highlight the importance of support during the discontinuation process, in particular psychosocial supports, and areas that could be targeted to improve the withdrawal process.

**Patient or Public Contribution:**

An international Steering Group was established to oversee and guide the PSP study in accordance with the James Lind Alliance guidance and Patient and Public Involvement principles. Patient and public groups were represented on the Steering Group and were among the three key stakeholder groups (i.e., people with lived experience of taking and/or stopping psychiatric medication, family members/carers/supporters and healthcare professionals) engaged with throughout.

## 1 Introduction

Global consumption of psychiatric medication has been increasing by approximately 4% annually, with the greatest increases observed with antidepressant and antipsychotic prescribing [1]. Rising prescription trends have led to concerns over the extent of the clinical improvement they provide in managing mental illnesses, as well as their potential for adverse effects and withdrawal symptoms [2, 3]. A longitudinal study reported that 30%–50% of antidepressant users may no longer be benefiting from continued treatment and should consider discontinuing [4]. Similarly, for hypnotic medication, sizeable numbers of the population are taking them for longer than recommended (i.e., > 4 weeks) [5].

There is a considerable cohort of individuals looking to reduce and/or discontinue long‐term use of psychiatric medication [6]. Reasons for discontinuing psychiatric medication include adverse effects associated with their use, the desire to recapture lost personal autonomy and to live a life free of medication [6, 7, 8, 9, 10]. There are several barriers to discontinuing psychiatric medication, which include withdrawal symptoms, relapse and availability of pharmaceutical dosage forms [11, 12, 13]. The uncertainties about what constitutes the optimal tapering approach and which interventions best support individuals discontinuing psychiatric medication also act as barriers to discontinuation [13, 14]. Tapering is the recommended approach for discontinuing psychiatric medication [15]. It involves gradually reducing the dose of the medication over a prolonged period [16, 17]. Tapering approaches vary in terms of the frequency and magnitude of dosage reductions [17]. Fixed dose tapering involves linear dose reductions in which the magnitude of the dose reduction remains the same throughout the tapering process [18]. Hyperbolic tapering involves dose reductions that follow a hyperbolic curve whereby the magnitude of dose reduction becomes smaller as the taper progresses based on the law of mass action and the hyperbolic dose–response that exists between drugs and receptors [18, 19]. However, there is currently a lack of robust evidence to inform guidance on tapering psychiatric medication, which has created many uncertainties about optimal tapering approaches and which interventions best support individuals discontinuing psychiatric medication [13, 14].

To address this, a James Lind Alliance (JLA) Priority Setting Partnership (PSP) study was conducted to identify the top 10 research priorities on reducing and stopping psychiatric medication [20]. The PSP study engaged key stakeholders representing people with lived experience of taking and/or stopping psychiatric medication, family members/carers/supporters and healthcare professionals. During the study, an anonymous online survey was shared with the stakeholder groups to gather key questions and uncertainties about the research topic. The survey also gave respondents the opportunity to submit additional free‐text comments. The aim of this paper is to report the descriptive analysis of respondents' views and experiences of reducing and/or stopping psychiatric medication use as documented in the free‐text responses submitted to this survey.

## 2 Methods

A qualitative descriptive analysis was undertaken of free‐text comments submitted to an anonymous online survey conducted as part of a PSP study to identify the top 10 research priorities on reducing and stopping psychiatric medication. Ethical approval was obtained from the Faculty of Health Sciences Research Ethics Committee, Trinity College Dublin (Ref: 220509).

### 2.1 The PSP Process

Full details of the PSP methodology and top 10 research priorities have been published separately [20]. Briefly, the PSP followed a seven‐step process, in line with the JLA recommendations, to enable the co‐production of research priorities. Initially, an international Steering Group (*n* = 13) representing the key stakeholder groups was established to guide and oversee the study. The Steering Group was also responsible for agreeing on the scope of the study (i.e., the breadth of the topic of interest). An anonymous online survey (Round 1) was conducted to gather stakeholders' uncertainties and questions about reducing/stopping psychiatric medication. In total, 884 survey respondents contributed 3635 questions, which were coded into eight major themes. Survey responses were summarised, and out‐of‐scope questions were removed. The remaining questions were checked against existing evidence, of which 32 questions were verified as uncertainties. These uncertainties were then ranked in a second online survey (Round 2) by 526 respondents. The top 10 priorities were determined during a 1‐day hybrid prioritisation workshop using a Nominal Group Technique involving 30 participants representing the key stakeholder groups.

### 2.2 Survey Development and Sampling

The Round 1 survey asked respondents to list their questions/uncertainties about the topic as free‐text responses. Respondents were asked for some brief demographic information and to select the stakeholder group that best represented them. The survey also included an optional additional comments section where respondents could add any additional comments about reducing and/or stopping psychiatric medication. There was no word limit on this section. The survey was accessed through Qualtrics, reviewed by the Steering Group and piloted by representatives from each stakeholder group. This paper focuses specifically on the analysis of the additional comments about reducing and/or stopping psychiatric medication from the Round 1 survey. No free‐text responses were collected in the Round 2 survey, which consisted of a shortlisting exercise of the identified priorities.

The survey was promoted using a multistrand snowball sampling approach, which included social media (e.g., Twitter/X), as well as newsletters and emails disseminated through organisations that had partnered with the study to support it (https://tapersafer.org/the-protect-study/our-partners). Steering Group members and study partners were asked to complete the survey and to share it with their networks. There are no formal target sample sizes for PSP surveys [21]. However, balanced stakeholder representation is desirable. Respondents' demographic profile was monitored, and several strategies were implemented to enhance engagement from the under‐represented stakeholder groups, which included targeted posts on Twitter/X.

To meet eligibility criteria, respondents had to be ≥ 18 years and represent one of the key stakeholder groups described above. There were no exclusion criteria based on geographical location or the use of other medications.

### 2.3 Data Collection

The survey link was included in all dissemination activities. Upon clicking the link, respondents were redirected to the study's website (www.tapersafer.org), where they could also access a Participant Information Leaflet and a narrated video with instructions on how to participate. Before commencing the survey, respondents were asked to self‐declare that they met the above eligibility criteria and consent to completing the survey voluntarily. The survey was entirely anonymous and was conducted between 4 November and 31 December 2022.

### 2.4 Data Analysis

All responses were downloaded into Microsoft Excel and then filtered based on stakeholder group. Each response was given a unique anonymous code which indicated whether the response was from the lived experience group (e.g., PLE0001, PLE0002), the family members/friends/carers/supporters group (e.g., FFCS0001, FFCS0002) or the healthcare professional group (e.g., HCP0001, HCP0002). The scope of the analysis was restricted to comments relating to tapering psychiatric medication (e.g., strategies used, associated challenges). Out‐of‐scope responses were removed from the final analysis (e.g., reasons for starting medication).

Data were analysed using template analysis, which is a technique for thematically organising and analysing qualitative data [22]. Following data familiarisation, a provisional coding template was developed and piloted on a subset of 50 responses by the researchers working independently. Coding was conducted by reviewing each response line by line. Coding was reviewed and discussed amongst team members to agree a final coding template, which was then applied to the remaining responses by two researchers (M.B., S.K.) working independently. Coding was compared between the researchers. Any disagreements throughout this process were resolved through discussion and input from a third researcher (C.C.). Once the data were coded in Excel, NVivo was then used to aid management of the coded data.

## 3 Results

### 3.1 Characteristics of Survey Respondents

In total, 80% (705/884) of respondents to the Round 1 survey completed the ‘Additional Comments’ section. Responses varied in length, ranging from a few sentences to several paragraphs. In total, 508 in‐scope responses were included in the final analysis after removal of out‐of‐scope responses (*n* = 197). Additional comments that were within scope were provided by all three stakeholder groups: people with lived experience of taking and/or stopping psychiatric medication (*n* = 389, 77%), healthcare professionals (*n* = 66, 13%) and family members/friends/carers/supporters (*n* = 53, 10%). Respondents primarily resided in the United States (42%), United Kingdom (21%) and Ireland (10%) with the remainder distributed across Europe (13%), Canada (6%), Oceania (5%), Africa (< 1%), Asia (2%) and Latin America (< 1%). Full details of respondents' demographics are reported in Table 1.

**Table 1:** Characteristics of Round 1 survey respondents.

	PSP Round 1 survey respondents	PSP Round 1 survey respondents who submitted additional comments	PSP Round 1 survey respondents who submitted in‐scope additional comments
	***n* (%)**	***n* (%)**	***n* (%)**
Total respondents	884	705	508
Gender			
Male	232 (26%)	175 (25%)	106 (21%)
Female	629 (71%)	518 (73%)	393 (77%)
Non‐binary	21 (2%)	10 (1%)	7 (1%)
Did not specify	2 (< 1%)	2 (< 1%)	2 (< 1%)
Stakeholder group			
Person with lived experience	609 (69%)	500 (69%)	389 (77%)
Family member, friend, carer, supporter	86 (10%)	80 (11%)	53 (10%)
Healthcare professional	186 (21%)	125 (18%)	66 (13%)
Other	3 (< 1%)	0	0
Age range, years			
18–24	22 (2%)	14 (2%)	10 (2%)
25–34	88 (10%)	58 (8%)	40 (8%)
35–44	164 (19%)	122 (17%)	82 (16%)
45–54	195 (22%)	161 (23%)	115 (23%)
55–64	236 (27%)	197 (28%)	153 (30%)
65–74	137 (15%)	118 (17%)	83 (16%)
75–84	39 (4%)	32 (5%)	22 (4%)
> 85	2 (< 1%)	2 (< 1%)	2 (< 1%)
Did not specify	1 (< 1%)	1 (< 1%)	1 (< 1%)
Continent			
Africa	15 (2%)	5 (< 1%)	3 (< 1%)
Asia	5 (< 1%)	5 (< 1%)	4 (1%)
Europe	394 (45%)	308 (44%)	194 (38%)
North America	420 (48%)	347 (49%)	273 (54%)
Oceania	41 (5%)	34 (5%)	28 (6%)
South America	7 (< 1%)	5 (< 1%)	5 (< 1%)
Did not specify	2 (< 1%)	1 (< 1%)	1 (< 1%)

### 3.2 Qualitative Findings

Six main themes were identified (Table 2): (1) experiences of psychiatric medication, (2) challenges to reducing/stopping psychiatric medication, (3) strategies used to reduce/stop psychiatric medication, (4) outcomes of reducing/stopping psychiatric medication, (5) emotional context and (6) areas for improvement.

**Table 2:** Overview of the key themes and subthemes relating to reducing and/or stopping psychiatric medication.

Themes	Subthemes
1: Experiences of psychiatric medication	I. Perceived benefits/harms of taking psychiatric medication
	II. Decisions around reducing/stopping psychiatric medication
2: Challenges to reducing/stopping psychiatric medication	I. Lack of support/recognition of withdrawal symptoms
	II. Lack of tapering resources
	III. Lack of autonomy
	IV. Withdrawal symptoms
3: Strategies used to reduce/stop psychiatric medication	I. Abrupt discontinuation
	II. Tapering
	III. Pharmacological supports
	IV. Non‐pharmacological supports
4: Outcomes of reducing/stopping psychiatric medication	I. Positive outcomes of reducing/stopping psychiatric medication
	II. Negative outcomes of reducing/stopping psychiatric medication
5: Emotional context	I. Anger and frustration
	II. Loneliness and hopelessness
	III. Gratitude and appreciation
6: Areas for improvement	I. Tapering resources and supports
	II. Regulatory oversight of the pharmaceutical industry
	III. Healthcare professionals' accountability/responsibility
	IV. Healthcare professionals' education and training
	V. Informed consent and public awareness

### 3.3 Theme 1: Experiences of Psychiatric Medication

This theme focused on respondents' experiences of psychiatric medication and was categorised into two subthemes.

#### 3.3.1 Perceived Benefits/Harms of Taking Psychiatric Medication

Several respondents reported benefiting from psychiatric medication. Perceived benefits included improved functioning, quality of life and recovery.

I am, a healthy, fit and socially active senior who is taking one medication that makes her life liveable. (PSP0324, PLE)

I think psychiatric medications are an important part of wellness and optimal functioning for anyone taking them.

(PSP0351, HCP)

Overall, respondents more commonly reported experiences of harm from taking psychiatric medication as opposed to benefits. Reported harms are primarily related to medication‐related adverse effects. Some healthcare professional respondents questioned the efficacy of psychiatric medication, their potential overprescribing and how they can negatively impact an individual's health and well‐being.

They don't work, lives are put on‐hold for years for withdrawal, and many are being left with life‐altering harms.

(PSP0002, FFCS)

I think they are often over prescribed and often there is no improvement in quality of life, yet the medication is not stopped and then goes on to have detrimental effects.

(PSP0503, HCP)

#### 3.3.2 Decisions Around Reducing/Stopping Psychiatric Medication

Adverse effects were the main reason that respondents from the lived experience group reduced/stopped their psychiatric medication. These ranged from headaches and nausea to suicidal ideation. In some cases, respondents felt that psychiatric medication was no longer needed or benefiting them.

I took an SSRI for 6 months, became increasingly depressed and suicidal, then tapered off.

(PSP0071, PLE)

I think most people stop psychiatric medication because of side effects. They feel meds make them feel fuzzy, out of it, hungover. Increased appetite and weight gain is a massive worry and problem for people and many stop meds because of weight gain.

(PSP1157, HCP)

Respondents from the lived experience group reported fear as a key barrier to reducing/stopping psychiatric medication. This included fear of the tapering process, withdrawal symptoms and relapsing. In contrast, a healthcare professional respondent reported that getting the service user to consider reducing/stopping psychiatric medication was the biggest barrier due to the skewed information they had received regarding the safety and efficacy of psychiatric medication.

I'm scared to start tapering again.

(PSP1086, PLE)

The public has been given a consistent message that antidepressants are the most effective (and convenient) way to address feelings of low mood. It can be a great challenge to explain the limitations of medication treatment… I find that addressing this challenge is the major difficulty in weaning patients off medication (rather than the tapering process itself).

(PSP0367, HCP)

### 3.4 Theme 2: Challenges to Reducing/Stopping Psychiatric Medication

This theme focused on the challenges to reducing/stopping psychiatric medication and was grouped into four subthemes.

#### 3.4.1 Lack of Support/Recognition of Withdrawal Symptoms

Many respondents from the lived experience and supporters' groups were dissatisfied with the availability and accessibility of tapering supports, particularly support from healthcare professionals. Some respondents also experienced reluctance from prescribers to reduce/stop psychiatric medication. Others reported difficulties in discussing withdrawal symptoms with prescribers included a lack of recognition or awareness of withdrawal symptoms.

The main problem for us has been finding a doctor who can support/advise about the whole process.

(PSP0429, FFCS)

Doing it my own way since I have zero help from my current doctors who look at me like I have two heads when I talk about withdrawals.

(PSP0259, PLE)

The reluctance amongst healthcare professionals to reduce/stop psychiatric medication was also discussed by some healthcare professional respondents, several of whom outlined potential justifications in their responses. These included uncertainty or lack of knowledge about the tapering process, lack of recognition for and underestimation of withdrawal symptoms and fear of criticism from other healthcare professionals.

The myth that people relapse if they are not on medication still informs the practice of most psychiatrists in my experience and they still believe the chemical imbalance hypothesis.

(PSP0496, HCP)

Fear of unfair criticism, ignorance of withdrawal and fear of admitting fault in assessing risks and benefits of meds are key to medical resistance to reducing and stopping meds.

(PSP0264, HCP)

#### 3.4.2 Lack of Tapering Resources

In the absence of dedicated evidence‐based tapering resources (e.g., guidelines), some respondents from the lived experience group sought information online from peer support websites and YouTube. Similar challenges were reported by healthcare professional respondents when making clinical decisions relating to the use and discontinuation of psychiatric medication, such as determining the risk–benefit ratio.

There is no science testing this, the drug companies make the pills tiny and incredibly hard to taper, and people are left to Googling YouTube videos and support sites for help.

(PSP0207, PLE)

The lack of clear guidelines makes it difficult to balance risk/benefit.

(PSP1293, HCP)

The lack of taper‐friendly formulations (i.e., liquids, low‐dose formulations) created practical issues for respondents from the three stakeholder groups in tapering psychiatric medication. These issues included difficulties with dose alterations and titrations to achieve desired medication doses. Healthcare professional respondents reported that it placed additional burdens on their already busy workloads.

I was struggling to cut my own dose in water because I couldn't cut the pill any smaller.

(PSP0108, PLE)

We have no access to tapering strips on the National Health Service and I have no time to support liquid reductions in clinical practice.

(PSP0345, HCP)

#### 3.4.3 Lack of Autonomy

Several respondents from the lived experience group reported struggles in having input in the decision‐making process relating to reducing/stopping psychiatric medication, whereby the prescriber was in full control of the taper. Some respondents who attempted to discuss tapering with their prescribers reported being dismissed and facing conflict.

My doctor is also pressuring me to complete my taper on HIS terms and schedule. I am being forced into a rapid taper by him.

(PSP1618, PLE)

#### 3.4.4 Withdrawal Symptoms

There was consensus across the three groups that the withdrawal process can be extremely difficult. Many respondents from the lived experience and supporters' groups experienced, or witnessed, withdrawal symptoms that appeared after reducing/stopping psychiatric medication, ranging from chronic pain and insomnia to suicidal ideation and brain fog. In some cases, the withdrawal symptoms were severe and persisted for prolonged periods after stopping psychiatric medication. Several respondents from the healthcare professional group acknowledged the potential severity of withdrawal symptoms and alluded to a general lack of understanding with respect to the underlying pathophysiology and management of withdrawal symptoms.

The hell endured in protracted withdrawal is the stuff of demonic nightmares.

(PSP1262, PLE)

Benzodiazepines are the least understood – the paradoxical sensitivity, the more difficult withdrawals on each attempt, how to respond to people who experience this hypersensitivity to withdrawal.

(PSP1528, HCP)

### 3.5 Theme 3: Strategies Used by Individuals to Reduce/Stop Psychiatric Medication

This theme focused on the strategies used by respondents to support them to reduce/stop psychiatric medications. The strategies were grouped into four subthemes.

#### 3.5.1 Abrupt Discontinuation

Several respondents had first or second‐hand experience of stopping psychiatric medication abruptly over a short period. In most cases, it was reported as being prescriber‐initiated. Abrupt discontinuation was only discussed by one healthcare professional respondent, who reported it to be the only option in some situations, given the lack of available pharmaceutical formulations.

When I went for help, all he did was give me a prescription that brought me down to zero in a week. That was an unsafe taper, too fast.

(PSP1092, PLE)

I have seen inpatients be abruptly stopped from psych meds when in ICU because there are no immediate release (crushable) forms available. Especially with duloxetine or other XR [extended release] medication. They just stop them! Maybe restart when they discharge.

(PSP0917, HCP)

#### 3.5.2 Tapering

Most respondents used or had witnessed a tapering strategy being used. These included reducing the medication dose by fixed amounts every few weeks and cutting tablets into smaller amounts. Some respondents switched from solid dosage forms to liquid formulations and then reduced the medication dose by calculating the amount of medication per drop. Many respondents reported that the tapering process took years to complete.

Venlafaxine ‐ took years to discontinue, fortunately managed in the end by cutting tablets into tiny portions and then increasing the length of time until the ‘electric shocks’ were unbearable and then taking another tiny portion.

(PSP0242, PLE)

I am still tapering diazepam using a liquid version. I'm taking 8 drops daily that equate to 0.4mg. I've tapered over 2 and 1/2 years from an initial amount of 80mg.

(PSP1424, PLE)

#### 3.5.3 Pharmacological Supports

Several respondents used or witnessed others using pharmacological supports. Examples included ketamine and marijuana. For some respondents, these pharmacological supports facilitated the withdrawal process and minimised the withdrawal symptoms. Other respondents reported switching to another psychiatric medication (i.e., ‘cross tapering’) before starting to reduce.

I use some limited [clonazepam] and [propranolol] just to get through events and responsibilities.

(PSP0098, PLE)

I have just swapped to a liquid form of a tricyclic medication before starting to reduce my meds.

(PSP1240, PLE)

#### 3.5.4 Non‐Pharmacological Supports

A smaller number of respondents used non‐pharmacological supports, which included diet and lifestyle changes, and cognitive behavioural therapy (CBT). Several respondents found that online peer support forums facilitated the withdrawal process by providing information, resources and support.

I try to eat clean whole foods and very limited processed foods. I exercise every day ‐ cycle, long walks, body pump, etc it really helps me.

(PSP1206, PLE)

I have experienced clients finding talking therapy adequate to facilitate their stopping medication.

(PSP0517, HCP)

### 3.6 Theme 4: Outcomes of Reducing/Stopping Psychiatric Medication

This theme focused on the outcomes of reducing/stopping psychiatric medication and was categorised into two subthemes.

#### 3.6.1 Positive Outcomes of Reducing/Stopping Psychiatric Medication

Many respondents had experienced or witnessed positive outcomes after reducing/stopping psychiatric medication. These ranged from a renewed sense of freedom and the ability to feel emotion and purpose, to relief from the adverse effects and suicidal thoughts linked to medication use.

Getting off the antipsychotic I was on has freed me from toxic psychiatry, brought more feeling, authentic emotion, more life, and more appreciation of life.

(PSP0428, PLE)

MOST patients become more socially and occupationally functional AFTER I reduce their polypharmacy.

(PSP0857, HCP)

#### 3.6.2 Negative Outcomes of Reducing/Stopping Psychiatric Medication

Respondents more commonly reported negative outcomes of reducing/stopping psychiatric medication compared to positive outcomes. These ranged from debilitating withdrawal symptoms and suicidal ideation to relapses requiring higher doses of the medication and reduced functional capacity, which had negative impacts on their personal and work life.

I lost 2 years of work, was tempted to take my own life several times and am still not healed completely.

(PSP0853, PLE)

He subsequently suffered seizures, etc., developed severe PAWS [Post Acute Withdrawal Syndrome] and then killed himself 4 months later.

(PSP0031, FFCS)

I have looked after numerous patients who have had catastrophic relapses after stopping their medication and it can be really difficult to get their symptoms back under control again, which often ends up with them being on more medication than they were before they stopped it.

(PSP1533, HCP)

### 3.7 Theme 5: Emotional Context

Respondents from the lived experience and supporters' groups experienced a diverse range of emotions upon, or after, reducing/stopping psychiatric medication. This theme was categorised into three subthemes.

#### 3.7.1 Anger and Frustration

Many respondents were angry and regretful about being put on psychiatric medication in the first place and for not being fully informed of the potential risks (e.g., physical dependence and withdrawal) before starting the medication.

I am angry that I have been put on this medication and not told what could happen to me.

(PSP0102, PLE)

For many, many times I wished I could go back in time and never lay my hands on any of them.

(PSP1279, PLE)

#### 3.7.2 Loneliness and Hopelessness

Several respondents reported feelings of loneliness and hopelessness while undertaking the withdrawal process. Some respondents feared that they had been permanently changed by taking psychiatric medication and that they would be on them for life.

Withdrawal is the worst, indescribable, socially isolating experience.

(PSP1295, PLE)

I had to do this all on my own, I lost everyone and everything important.

(PSP0062, PLE)

I hate them and feel trapped to take them for the rest of my life.

(PSP0193, PLE)

#### 3.7.3 Gratitude and Appreciation

Respondents from the three groups expressed their gratitude and appreciation to the research team and its collaborators for conducting research into this topic and for the opportunity to participate.

Thank you so much for your work on the behalf of people suffering from psychotropic drugs and their withdrawal.

(PSP0555, PLE)

Just to give thanks to your efforts and remark the importance of this topic.

(PSP0310, HCP)

### 3.8 Theme 6: Areas for Improvement

This theme focused on potential areas that could be targeted to improve individuals' experiences of the withdrawal process and was categorised into five subthemes.

#### 3.8.1 Tapering Resources and Supports

Respondents from the three groups suggested that evidence‐based and accessible tapering guidelines should be developed, and tapering supports should be improved. Respondents believed that tapering guidelines have the potential to facilitate decision‐making, guide the tapering process and minimise withdrawal symptoms. Respondents also asked for more taper‐friendly formulations (i.e., lower dose formulations and tapering strips) to overcome the practical issues of manipulating solid dose formulations discussed above.

It would be great to have better, more up‐to‐date evidence‐based guidelines to help clinicians like her make recommendations to patients like me.

(PSP1640, PLE)

There should be lower doses readily available without using expensive compounding pharmacies. The companies who make the drugs should also make lower doses to help people taper.

(PSP0302, PLE)

Respondents across the three groups also asked for improved tapering supports. These included financial supports in the form of medical insurance/government assistance during and after the withdrawal process, psychosocial supports in the form of online supports and residential care and improved availability of trained healthcare professionals in primary care.

More supports need to be mandated, so that mental health patients get the protections they need, when faced with difficult situations around medications, while being forced to work with flawed practitioners.

(PSP1578, PLE)

We need realistic care pathways/interventions that can be implemented in primary care so we can best support the large numbers of people who wish to reduce or stop psychotropic medicines. High‐intensity interventions needing specialist staff will only ever be available to a small number of patients.

(PSP1536, HCP)

#### 3.8.2 Regulatory Oversight of the Pharmaceutical Industry

Respondents from the lived experience and supporters groups queried the level of regulatory oversight of psychiatric medication, and the level of accountability and responsibility of the pharmaceutical industry for the harms caused by these medications. Several respondents asked for enhanced regulatory oversight of the prescribing of psychiatric medication (e.g., two‐person prescribing) and questioned the relationship between the pharmaceutical industry and prescribers.

Why are the pharmaceutical companies not accountable for the damage they have caused to so many.

(PSP0471, PLE)

These should all be highly controlled drugs and require two doctor signatures of agreement that it is a correct option.

(PSP0062, PLE)

#### 3.8.3 Healthcare Professionals' Accountability/Responsibility

Several respondents from the lived experience and supporters groups queried the level of accountability and responsibility of healthcare professionals in areas relating to information provision, shared decision‐making and prescribing of psychiatric medication. Others queried the reluctance amongst healthcare professionals to reduce/stop psychiatric medication and how this could be overcome, suggesting that they be mandated to stop psychiatric medications when the risks outweigh the benefits. Although respondents from the healthcare professional group raised similar questions, they appeared unclear as to what information they should be providing individuals with upon initiating psychiatric medication.

How to MAKE prescribers warn patients about the addictive nature of these medications at the start of ‘treatment’. And help patients understand what this will mean for their future.

(PSP0394, FFCS)

Should we be talking about patients about some of the issues re: stopping psychiatric medicines when we start them on then? So that they are fully informed.

(PSP1529, HCP)

How could the doctors who prescribed the drugs now causing insufferable side effects and often even worse withdrawal effects be brought face to face with the suffering caused by their practice of medicine?

(PSP0554, HCP)

#### 3.8.4 Healthcare Professionals' Education and Training

Respondents from the lived experience and supporters groups perceived the level of tapering knowledge amongst healthcare professionals as inadequate. The only response from a healthcare professional linked to this subtheme shared these views, whereby the respondent acknowledged the lack of tapering‐related education they had received during their training as a pharmacist.

My pharmacist has no clue. My primary care physician has no clue, and even my psychiatrist had no clue.

(PSP0325, PLE)

Pharmacists were told nothing! All I knew about benzos from school was if you stop too fast you can have seizures.

(PSP0917, HCP)

Respondents from the three groups asked for enhanced training of healthcare professionals to address their perceived knowledge gaps surrounding psychiatric medication, specifically reducing/stopping psychiatric medication. Some suggested that this training be mandatory. It was postulated that enhanced education may overcome challenges relating to informed consent. According to one healthcare professional respondent, legal changes restricting prescription quantities and durations are needed alongside education.

Do everything to education doctors and hospice organizations to in turn thoroughly seriously warn their patients that you can be hooked in two weeks and take over a year to taper and you may not succeed.

(PSP0021, FFCS)

The problem with psychotropic drug withdrawal is enormous. It needs a multiprong approach, education: health care providers and students of these disciplines and to the public, legal: with prescribing restrictions for amount and duration.

(PSP0991, HCP)

#### 3.8.5 Informed Consent and Public Awareness

Several respondents from the lived experience and supporters groups asked for enhanced information provision at the individual service user level, including a greater emphasis on the difficulties of reducing/stopping psychiatric medication. In their view, this may improve service users' decision‐making capacity and their ability to give informed consent.

I wish it were more often told to people BEFORE they are prescribed these medications how HARD it is to get off them.

(PSP0044, PLE)

Difficulties with reducing and stopping must be part of informed consent before people start psychoactive medication.

(PSP0481, FFCS)

Respondents from the three groups also asked for enhanced information provision around the potential harms associated with the use and discontinuation of psychiatric medication at a wider, societal level to other groups, such as the public and family members. One respondent from the lived experience group perceived a need for a public conversation to remove the stigma around reducing/stopping psychiatric medication, which contrasted with views held by some respondents from the healthcare professional group. One respondent asked for a less polarised discourse around reducing/stopping psychiatric medication because, in their view, the current discussion was anti‐medication. While another respondent asked for a more balanced approach when discussing psychiatric medication because, in their opinion, psychiatric medication is a controversial issue, and it is easy to end up on one side.

Please make society aware that is ok and natural to stop/end the psychiatric drugs and not take them permanently for every human being who had a hard time or reached another state of consciousness!

(PSP0144, PLE)

There needs to be more information available to these families that are effected.

(PSP0123, FFCS)

I would like to see less demonization of psychotropic medications in this discourse around reducing and stopping ‐ most patients would benefit more from stopping tobacco, alcohol and other substances than from discontinuing psychotropics.

(PSP0010, HCP)

## 4 Discussion

This study examined the views and experiences of reducing and/or stopping psychiatric medication among a sample of respondents to a survey conducted as part of a PSP study [20]. These respondents represented people with lived experience of taking and/or stopping psychiatric medication, family members/carers/supporters and healthcare professionals. For many respondents from the lived experience group, the withdrawal process was a long and arduous journey. In their responses, respondents discussed their experiences of various parts of the withdrawal process. Although respondents from the supporter and healthcare professional groups offered different perspectives, there was a high level of concordance between the three stakeholder groups on many issues across all major themes. This study also highlighted respondents' suggestions on how the withdrawal process could potentially be improved.

Adverse effects were the main reason that respondents from the lived experience group reduced/stopped their psychiatric medication. This aligns with a recent longitudinal study involving antidepressant users, which found that 20% of those who stopped treatment did so because of adverse effects [23]. Fear was a key barrier that respondents reported to reducing/stopping psychiatric medication (i.e., fear of relapse and withdrawal symptoms). In an interview study involving antipsychotic users, 70% of those who did not want to discontinue antipsychotics cited fear of relapse as the reason [9]. Few respondents from the healthcare professional group elaborated on reasons for reducing/stopping psychiatric medication in their responses, which made it difficult to compare their views on this issue.

Most respondents from the lived experience group reduced/stopped their psychiatric medication by tapering the dose. However, the reported scarcity of information and guidelines created challenges with identifying and employing appropriate tapering methods. Despite the recent development of tapering guidance by organisations such as the National Institute for Health and Care Excellence (NICE) and the UK's Royal College of Psychiatrists [24], there is a pressing need for further comprehensive guidelines to support individuals looking to reduce and/or stop psychiatric medication [14, 25].

Many respondents also reported practical challenges when following a tapering regimen which called for minor dose adjustments. These included difficulties with dose alterations and titrations to achieve desired medication doses. Respondents attributed these practical issues to the lack of taper‐friendly formulations (i.e., tapering strips, liquid formulations). While these issues were previously identified by a survey involving individuals stopping antidepressants [26, 27] and emerged as one of the research priorities in the PSP study [20], what is novel about these study findings is that they also captured the impact of these issues on respondents and their attempts to overcome them. For example, respondents cut tablets or switched to liquid formulations and then reduced the dose. A recent systematic review highlighted limited published research investigating the accuracy of different methods of manipulating psychotropic medications, which needs to be addressed to better inform the tapering approaches that are used in real‐world settings [28].

Many respondents from the lived experience group reported difficulties in finding a supportive healthcare professional who would guide the withdrawal process and recognise the adverse effects and challenges associated with the use and discontinuation of psychiatric medication. Studies involving people with lived experience have highlighted similar challenges around professional support [29]. Previous research has also identified reluctance amongst clinicians to support individuals wishing to taper antipsychotics [30]. This reluctance may have contributed to the lack of autonomy experienced by several respondents from the lived experience and supporters' groups in terms of having an input in the decision‐making process. Potential justifications proposed by healthcare professional respondents were fear of criticism by their colleagues, lack of high‐quality evidence underpinning the process of withdrawal and the knowledge gaps that have been created as a result. Other respondents whose medication had been forcibly stopped by their prescriber regarded this as a threat to their autonomy. The World Health Organisation (WHO) promotes shared decision‐making and informed consent as crucial components to the provision of person‐centred and recovery‐oriented care [31]. The importance of shared decision‐making has also been recognised by healthcare professionals [32]. Despite this, service users have previously reported challenges in having their autonomy and choice respected and being involved in decisions around their medication [33]. This feeds back into the traditional paternalistic approach to mental healthcare, one in which the prescriber yields all the power. It does not align with the more modern, rights‐based, recovery‐oriented approach to mental healthcare that seeks to promote open discussion about medication [31].

The use and discontinuation of psychiatric medication was an emotive topic for many respondents from the lived experience and supporters' groups, provoking a range of emotions, which included anger, loneliness and hopelessness. Previous studies involving individuals who had stopped psychiatric medication have also reported negative emotions regarding the use of psychiatric medication, including a sense of regret over taking the medication in the first instance, as well as fear and worry, which acted as barriers to subsequently stopping the medication [34, 35, 36, 37].

Although some respondents reported positive outcomes after reducing/stopping psychiatric medication, such as functional and social improvements, several negative outcomes were also reported, including relapses and protracted withdrawal symptoms. Despite previous clinical guidelines which stated that withdrawal symptoms were unlikely to persist for a prolonged period after stopping psychiatric medication, such as antidepressants [38], more recent evidence has highlighted that withdrawal symptoms can persist for months or years after reducing or stopping the medication [39, 40]. When this happens, acute withdrawal transitions into a protracted withdrawal syndrome [39, 40].

Some respondents made suggestions on how the above challenges could be addressed with a view to improving the withdrawal process. Enhanced regulatory oversight of the pharmaceutical industry was another area that was mentioned exclusively by respondents from the lived experience group. Specific examples included tighter controls of prescriptions for psychiatric medications and enhanced oversight of the relationship between the pharmaceutical industry and prescribers. Similar issues were identified by a study involving individuals discontinuing benzodiazepine receptor agonists [29]. Respondents also called for better education and training of healthcare professionals, and increased accountability and responsibility for their actions. This recommendation was strengthened by respondents from the healthcare professional group who acknowledged the lack of education they had received and asked to be enrolled on tapering‐related training programs. A previous study also identified important perceived deficits in healthcare professionals' knowledge of psychiatric medication and tapering approaches among individuals taking psychiatric medication [41]. According to a survey involving individuals who had stopped antidepressants, the most frequently cited recommendation for future withdrawal services was to improve healthcare professional knowledge [26].

There was an immense sense of gratitude and appreciation expressed by respondents from the three stakeholder groups for the opportunity to contribute to this research and to have their voices heard. Traditionally, mental health research excluded individuals who had received a psychiatric diagnosis from participating in research [42]. By regarding respondents from the stakeholder groups as equals, this study gave all individuals the same opportunity to share their experiences of taking and/or stopping psychiatric medication, and aligned with the more modern, rights‐based approach to mental health research [43].

### 4.1 Strengths and Limitations

The major strength of this study was its inclusion of a diverse sample of responses comprising different stakeholder groups (people with lived experience of taking and/or stopping psychiatric medication, family members/carers/supporters and healthcare professionals) across different countries. This allowed for a wide range of views and experiences to be captured whilst illustrating the diverging views and experiences that exist in mental health research around psychiatric medication use. In terms of study limitations, the responses were heavily dominated by the lived experience group. As a result, it was not always possible to make comparisons with the other stakeholder groups. In keeping with the JLA approach, respondents were not asked to provide personal information about their health status or which medications they were taking or had previously taken. As a result, it was not possible to provide further information about the sample or comment on which medications were represented more than others. The reliance on voluntary completion of the survey created the potential for self‐selection bias, as individuals with more negative experiences of discontinuing psychiatric medication may have been more inclined to complete the survey than those who did not. The use of an online survey and the need to provide written responses may also have impacted the profile of respondents. For example, there is a recognised risk of digital exclusion among people with severe mental illness due to a lack of relevant digital skills [44]. Previous research has also shown that they may also struggle with reading and expressing themselves in written form [45]. To enhance accessibility and engagement with this research, the surveys were piloted by representatives of the lived experience group and the written study information was supplemented with a narrated video, which guided respondents through the survey to assist them with its completion. It must also be noted that there are several active online communities and discussion boards dedicated to supporting individuals experiencing difficulty with discontinuing psychiatric medication, which rely on written text [17, 46]. Consequently, although the findings cannot claim to be generalisable to all patients discontinuing psychiatric medications, they provide novel and important insights into the experience of a particular cohort of those that have experienced difficulties. Finally, the reliance on free‐text response data risked interpretative bias during the coding process; hence, all data were coded by at least two researchers.

### 4.2 Conclusion

This study identified numerous challenges faced by individuals upon withdrawing psychiatric medication and the uncertainty that prevails in terms of the best tapering strategy. The findings highlight the importance of support during the withdrawal process, in particular non‐pharmacological supports, and suggest areas that could be targeted to improve the withdrawal process. The study also provided important and novel insights into the human and emotional impact of taking and stopping psychiatric medication. These findings may encourage a broader discussion about the role of these medications in treating mental illness and when to start or stop treatment.

## Author Contributions

**Miriam Boland:** conceptualisation, funding acquisition, methodology, investigation, formal analysis, project administration, writing – original draft preparation. **Agnes Higgins:** conceptualisation, funding acquisition, methodology, investigation, formal analysis, writing – review and editing. **Sookyung Kwak:** formal analysis, writing – review and editing. **Cathal Cadogan:** conceptualisation, funding acquisition, methodology, investigation, formal analysis, writing – review and editing. Cathal Cadogan acted as guarantor.

## Conflicts of Interest

C.C. sits on the board of directors for the Alliance for Benzodiazepine Best Practices, a not‐for‐profit organisation with the mission to inform evidence‐based improvements in the use of benzodiazepines and Z‐drugs. All other authors declare no competing interests.

## Data Availability

Data are available upon reasonable request.
